# Nutritional status of children and adolescents with Type 1 Diabetes Mellitus in Baghdad: a case-control study

**DOI:** 10.25122/jml-2022-0233

**Published:** 2023-02

**Authors:** Sawsan Ali Hussein, Basma Adel Ibrahim, Wasnaa Hadi Abdullah

**Affiliations:** 1 Pediatric Department, College of Medicine, Mustansiriyah University, Baghdad, Iraq

**Keywords:** anthropometric measurements, children, nutritional status, type 1 diabetes mellitus, undernutrition

## Abstract

Diabetes mellitus (DM) is a major life-long non-communicable illness correlated with obesity and chronic undernutrition. It is particularly important to monitor the nutritional status of children with type 1 diabetes mellitus (T1DM), as they are still growing and may be affected by the disease or associated conditions like celiac disease. This study aimed to evaluate the nutritional status of children and adolescents with T1DM in Baghdad city and identify possible risk factors for undernutrition. A single-center, case-control study was conducted in Central Child's Teaching Hospital, Baghdad, Iraq, over 9 months from November 2021 to July 2022. The study included patients with T1DM and healthy controls. Detailed history, clinical examination, and anthropometric measures were performed for all participants in the study. The mean age of the sample was 10.0 ±3.73 years and 8.68±3.1 years in diabetic patients and controls, respectively. Anthropometric measures in patients with type 1 diabetes were significantly lower than those of controls (P<0.001). All patients within the undernourished group were from large-size families compared with 75.76% of the normally nourished group, with a significant difference. The mean age of disease onset in the normal nourished group was 6.61 ± 2.78 years which was significantly earlier than that of the undernourished group (8.83 ± 2.89). Weight-for-age and BMI z-score had a significant negative correlation with HbA1c (r=-0.312, p=0.004, and r=-0.295, p=0.006, respectively). Patients with T1DM had significantly lower anthropometric measures than the normal population. Older children, female gender, large family size, and disease duration are independent predictors of undernutrition in T1DM. BMI and weight-for-age have a significant negative correlation with metabolic control of diabetes represented by HbA1c.

## INTRODUCTION

Diabetes mellitus (DM) is a major life-long non-communicable illness that has been correlated with both obesity and chronic undernutrition [1,2]. Type 1 DM was previously listed as one of the causes of severe growth retardation. In Iraq, the healthcare system has been disrupted by wars and conflicts that affect health services, affecting the glycemic control of children living with diabetes [3]. Diet is a core component of type 1 diabetes management, and poor diet quality may affect metabolic control and other health outcomes. However, there is a lack of research on diet quality in children and adolescents with DM [4]. Nutritional status should be regularly evaluated in type 1 DM children as they are still growing and are at risk of malnourishment due to the chronic and debilitating nature of the illness or the presence of associated celiac disease [5]. Factors that affect growth in DM patients include gender, genes, age at onset, growth hormone levels, disease duration, metabolic control, and puberty [6]. Pediatric undernutrition is described as a disproportion between nutrient demand and intake, leading to accumulative deficits of protein, energy, and micronutrients that may have negative effects on their growth and normal development [7]. It is a substantial issue in children with type 1 DM, and evaluation of growth should be considered during the regular follow-up visits of these patients [8]. Evaluation of undernutrition demands anthropometric measurements of body weight and length/height and plotting these variables on population growth charts for comparison against normal values [9]. However, there is an ongoing debate about the most useful measurement in DM follow-up and the consistency of anthropometric parameters. Therefore, a combination of measurements, including body weight, height, mass index, MUAC, and TSFT, should be considered along with other clinical parameters for nutritional evaluation in DM children [7]. This study aimed to evaluate the nutritional status of children and adolescents with type 1 diabetes mellitus in Baghdad city and identify possible risk factors for undernutrition.

## MATERIAL AND METHODS

A case-control study was conducted at Central Child's Teaching Hospital in Baghdad, Iraq, over 9 months from November 1st, 2021, to July 31st, 2022. The study included 84 randomly selected patients with type 1 diabetes mellitus, between the ages of 3 and 18, who were under insulin treatment and had a diabetes duration of at least 1 year. They were compared to 84 age and gender-matched healthy controls. Patients with type 2 diabetes and those with chronic conditions such as celiac disease, hypothyroidism, inflammatory bowel diseases, or any other medical syndromes were excluded from the study.

### Sample size

The sample size was calculated according to the following formula: N=Z2P(1−P)/d2, where N represents the sample size, Z corresponds to the level of confidence, P is the expected prevalence, and d represents the precision (corresponding to effect size) [10]. According to previous studies that reported a prevalence of T1DM of approximately 5% among Iraqi children, the sample size was calculated as follows:


$$N={\left(1.96\right)}^{2}\times \;\left(0.05\right)\;\times \;\left(1-0.05\right)\;/\;{\left(0.05\right)}^{2}=73$$

### Questionnaire

Data were collected using a standardized questionnaire that included age (divided into three categories: preschool [<6 years], school age [6-12 years], and adolescent [13-18 years]), sex, family size (defined as families with three or more children considered as large and those with ≤ 2 children regarded as small) [8], family income (classified as low income [< 100,000 ID per capita], medium income [100,000- 250,000 ID per capita], and high income [> 250,000 ID per capita]) [8]. In addition, for the group of patients with diabetes, collected data included age at the onset of diabetes, duration of diabetes since diagnosis, number of diabetic ketoacidoses throughout the illness, and the most recent HbA1c test result (taken within 3 months). HbA1c values were measured using a direct enzymatic assay and were categorized as good (less than 7.5%), intermediate (7.5% to 9.0%), or poor (more than 9.0%) glycemic control following the International Society for Pediatric and Adolescent Diabetes website [11].

### Anthropometric parameters

All patients underwent a general and systematic evaluation, including the assessment of anthropometric parameters such as weight, height, body mass index (BMI), triceps skin fold thickness (TSFT), and mid-upper arm circumference (MUAC). Weight and height measurements were taken with participants wearing light clothing and no shoes. Weight measurements were taken on a manual scale, with the participant standing on the scale with arms extended along the side of the body and the researcher ensuring the back was straight. Height was measured with a wall stadiometer with a 0.1 cm precision scale. Height was measured with a wall stadiometer with a 0.1 cm precision scale, with the participant standing barefoot with their back to the wall [12]. Body mass index (BMI) was calculated by dividing weight in kilograms by the square of height in meters [13]. The mid-upper arm circumference was measured using a Gulick tape (Baseline 12-1201) with an accuracy of 0.5 cm, following the guidelines of the National Health and Nutrition Examination Survey [12]. Furthermore, the triceps skin fold thickness was measured using a Saehan Medical Skinfold Caliper (SH5020) with 0.1 mm accuracy. Measurements were taken just below the shoulder blades in a horizontal grip, just above the triceps muscle of the upper arm vertical grip, and on the stomach level in an oblique grip at a quarter the distance between the navel and the iliac on the non-dominant side of the body. The test was repeated 3 times from each location, and then the average was calculated from the results obtained [14]. The growth parameters were corrected for age by converting them to z scores [15]. Z scores were utilized because they allow more accuracy in expressing anthropometric status than traditional placement “near” or “below” a certain percentile curve [7]. Normal measures are within 2 standard deviations (SD) from the mean, while those > 2 SD below the mean indicate malnutrition [16].

### Statistical analysis

Statistical analysis was performed using SPSS software version 25.0 (SPSS, Chicago, USA). The normality of data was tested using the Shapiro-Wilk test, and normally distributed data were presented as mean ± SD. Comparisons for continuous data were made using a Student’s t-test. Data with non-normal distribution were described as median and range and were analyzed using the Mann-Whitney U test. Categorical data were expressed in numbers and percentages and analyzed using a Chi-square/Fischer exact test. The correlation between different national indices with age and disease duration was explored by Pearson’s correlation. Multivariate logistic regression was used to identify independent predictors of undernutrition in T1DM patients according to BMI. A p-value less than 0.05 was considered statistically significant.

## RESULTS

In the current study, the mean age of patients with diabetes and controls was 10.0 ± 3.73 and 8.68 ± 3.1 years, respectively, with no significant difference (P= 0.069). Among patients with diabetes, 63.1% were in the school-age group (6-12 years), 14.29% were adolescents (13-18 years), and 9.52% were below 6 years old. Females represented 65% and 75% of patients and controls, respectively, with no significant difference. Low income was reported in more than half (54.67%) of the patient's families, and the large family size was prevalent among the majority (80.95%) of patients. The mean age at onset and disease duration was 7.08±2.93 years and 3.0±2.61 years, respectively, with 76.19% of the patients having a disease duration of less than 5 years. Most patients (76.19%) experienced DKA 0-2 times, while only a minority (4.67%) experienced such complications 6-8 times throughout their illness. The HbA1c level was markedly elevated, with a mean of 10.26±2.31%, and 11.9%, 26.19%, and 61.9% of patients had HbA1c levels of <7.5, 7.5-9, and >9, respectively. The anthropometric measurements in patients and controls are shown in Table 1, with the weight for age, height for age, BMI, and MUAC z scores significantly lower in patients with T1DM than in controls. However, there was no significant difference between the two groups in the TSFT z score.

**Table 1:** Anthropometric measurements in diabetic patients and controls.

Variables	Patients (n=84)	Controls (n=84)	P-value
**Weight for age z score**			
Mean±SD	-0.28±0.13	0.62±1.23	<0.001
Median	-0.45	0.57	
Range	-2.87-2.55	-2.0-3.0	
**Height for age z score**			
Mean±SD	-0.66±1.29	0.27±1.3	<0.001
Median	-0.75	0.32	
Range	-3.14-2.37	-3.0-3.7	
**BMI for age z score**			
Mean±SD	0.13±1.2	2.19±0.96	<0.001
Median	0.09	2.33	
Range	-2.25-2.32	-0.36-3.8	
**Mid upper arm circumference z score**			
Mean±SD	-1.53±1.37	1.57±2.38	<0.001
Median	-1.5	1.0	
Range	-4.86-1.39	-1.92-7.0	
**Triceps skinfold thickness z score**			
Mean±SD	0.51±0.94	0.64±1.15	0.282
Median	0.51	0.75	
Range	-2.51-2.43	-2.0-2.7	

Wasting, defined as weight > 2 SD below the mean for age, and short stature, defined as height > 2 SD below the mean for age [17], were observed in 18 (21.42%) and 32 (38.09%) patients with diabetes, respectively. Low BMI was found in 18 (21.42%) patients with diabetes. Table 2 shows the associations between demographic and clinical characteristics of the patients with BMI z score. Adolescent patients were more common among the undernourished group, and the reverse was true for school-age patients, with a highly significant difference. All patients within the undernourished group were from large-size families compared with 75.76% of the normal nourished group, with a significant difference. The mean age of disease onset in the normal nourished group was 6.61 ± 2.78 years which was much earlier than that of the undernourished group (8.83 ± 2.89) with a highly significant difference. However, the patient' s gender, family income, disease duration, number of DKA episodes, and HbA1c levels were not different in the well-nourished and undernourished groups.

**Table 2:** Association of demographic data and clinical characteristics of patients with BMI z score.

Variables	Normal (n=66)	Undernourished (n=18)	P-value
**Age, years**			
Preschool (<6)	6 (9.09%)	2 (11.11%)	0.007
School-age (6-12)	47 (71.21%)	6 (33.33%)	
Adolescent (13-18)	13 (19.70%)	10 (55.56%)	
**Gender**			
Male	28 (42.42%)	4 (22.22%)	0.118
Female	38 (57.58%)	14 (77.78%)	
**Income**			
Low	22 (33.33%)	10 (55.56%)	0.197
Medium	42 (63.64%)	8 (44.44%)	
High	2 (3.03%)	0 (0%)	
**Family size**			
Small	16 (24.24%)	0 (0%)	0.020
Large	50 (75.76%)	18 (100%)	
**Age at onset, years**			
Mean±SD	6.61±2.78	8.83±2.89	0.004
**Disease duration, years**			
<5	48 (72.72%)	16 (88.89%)	0.154
≥5	18 (27.28%)	2 (11.11%)	
**Diabetic ketoacidosis, No.**			
0-2	52 (78.79%)	16 (88.89%)	0.148
3-5	10 (15.15%)	2 (11.11%)	
6-8	4 (6.06%)	0 (0%)	
**HbA1c, %**			
<7.5	8 (12.12%)	2 (11.11%)	0.738
7.5-9	16 (24.24%)	6 (33.33%)	
>9	42 (63.64%)	10 (55.56%)	

A multivariate logistic regression test was performed to determine the independent predictors of undernutrition in T1DM patients based on BMI. All variables with a p-value of ≤0.150 in the univariate analysis (shown in Table 3) were included in the model. The results revealed that school age (OR=0.42, 95%CI= 0.12-0.87, p= 0.031), adolescent (OR= 3.17, 95% CI= 1.14-32.54, p= 0.019), female gender (OR= 2.6, 95% CI= 1.08-28.45, p= 0.048), family size ≥5 members (OR= 3.22, 95%CI= 1.23-22.98, p= 0.011) and disease duration (OR= 2.8, 95%CI=1.18-19.67, p= 0.032) were independent predictors of undernutrition in T1DM patients according to BMI (Table 4).

**Table 3:** Correlation of type 1 DM duration and HbA1c with growth parameters.

Variables	Disease duration		HbA1c	
	Correlation	P-value	Correlation	P-value
**Weight for age z-score**	0.107	0.334	-0.312	0.004
**Height for age z-score**	-0.020	0.859	-0.164	0.136
**BMI z-score**	0.045	0.683	-0.295	0.006
**TSFT z-score**	-0.102	0.331	0.141	0.201
**MUAC z-score**	-0.149	0.177	0.145	0.190

**Table 4:** A list of predictors of undernutrition in type1 DM patients according to BMI.

Variables	Odds ratio	95% Confidence Interval	P-value
**Age, years**			
Preschool (<6)	1.0		0.031
School-age (6-12)	0.42	0.12-0.87	0.022
Adolescent (13-18)	3.17	1.14-32.54	0.019
**Gender**			
Male	1.0		0.048
Female	2.6	1.08-28.45	
**Family size**			
Small	1.0		0.011
Large	3.22	1.23-22.98	
**Disease duration, years**			
<5	1.0		0.032
≥5	2.8	1.18-19.67	
**Diabetic ketoacidosis, No.**			
0-2	1.0		0.126
3-5	0.88	0.12-2.65	0.238
6-8	0.67	0.22-4.32	0.432

**Figure 1:**
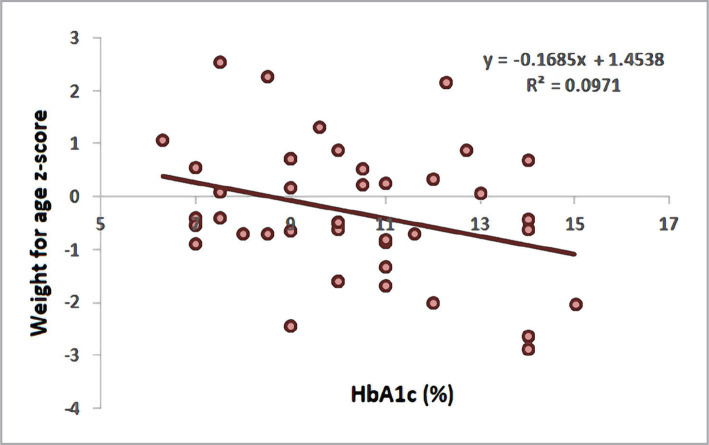
Scatter plot and regression line between HbA1c and weight-for-age z-score.

**Figure 2:**
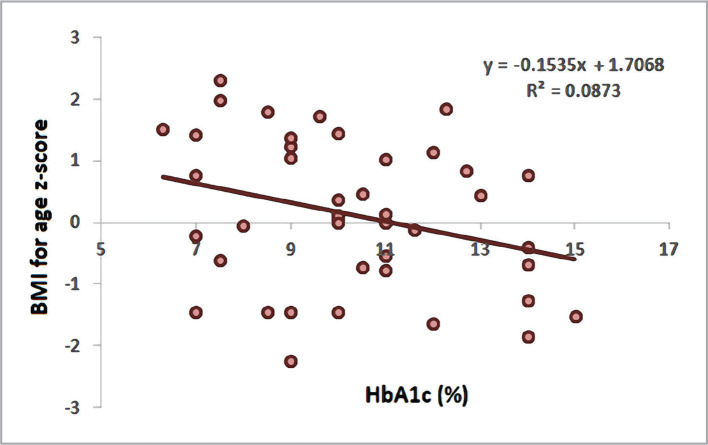
Scatter plot and regression line between HbA1c and BMI for age z-score.

Weight-for-age z-score revealed a significant negative correlation with HbA1c (r = -0.312, p = 0.004). Furthermore, the BMI z-score also displayed a significant negative correlation with HbA1c (r = -0.295, p = 0.006), as shown in Table 4, Figures 1 and 2.

## DISCUSSION

Few studies have focused on assessing the nutritional status of children and adolescents with type 1 DM. The current study emphasizes the importance of periodically evaluating anthropometric parameters and assessing the nutritional status of these patients. This provides a safe, non-invasive means for medical professionals to monitor metabolic control and ensure that type 1 DM patients achieve typical somatic development for their age group [17]. Our findings revealed higher rates of wasting and short stature (21.42% and 38.09%, respectively) among diabetic patients compared to previous studies by Khadilkar *et al*. [18] in India and Aljuhani *et al*. [6] in Saudi Arabia, which reported lower rates of 10.9% and 27.1%, and 6.9% and 11.9%, respectively. Variations in sample size and population characteristics may account for these differences.

The anthropometric measures, including weight for age, height for age, BMI, and MUAC z-scores, were significantly lower in diabetic patients compared to controls, which is consistent with previous studies such as Dohan *et al*., [8] in Iraq, Khadilkar *et al*., [18] in India, and Stipancić *et al*., [19] in Croatia, suggesting that diabetes has a negative impact on nutritional status. Furthermore, this study found that malnourished patients with diabetes were mostly adolescents, and older age at disease onset was significantly associated with malnutrition. This finding may be explained by the fact that older age groups may experience dietary restrictions, while younger age groups may not be as restricted in their diet, which is crucial for their normal growth and development. In addition, the effect of sex steroids on adolescents may counteract the effect of insulin, potentially leading to the derangement of their metabolic control. Regarding the number of family members, this study found that large family size was significantly associated with low BMI in individuals with diabetes, which could be explained by a decrement in nutritional and family care with an increase in the number of siblings. The earlier study by Dohan *et al*. [8] found a non-significant correlation which could be attributed to variation in sample size.

The present study identified several independent risk factors for undernutrition in type 1 diabetes, including school age, adolescent age, female gender, large family size, and longer disease duration. These findings are supported by previous studies, including Dohan *et al*. [8] and Mousa *et al*. [20], which also found undernutrition more common in females. However, Hassan *et al*. [21] found no significant correlation between gender and growth parameters. Bonfig *et al*. [22] noted a negative correlation between adult height and the duration of diabetes in a large study of over 22,000 children with type 1 DM in Germany and Austria, while Aljuhani *et al*. [6] found no significant effect of gender or disease duration on undernutrition. The correlation between all growth measures in diabetic patients and HbA1c showed a significant negative correlation between weight for age and BMI with HbA1c, which is consistent with the findings of Aljuhani *et al*. [6] and Dohan *et al*. [8]. Hassan *et al*.[21] found a negative correlation between metabolic control and all anthropometric measures, while the study by Khadilkar *et al*. [18] from India and Galli-Tsinopoulou *et al*. [23] from Greece observed no correlation between HbA1c and growth parameters. This could be attributed to the variation in sample size and general characteristics of the population. The correlation between glycemic control or the duration of illness and child growth varies among studies. Thus, conducting a systematic review of these studies may be crucial in gaining a deeper insight into the growth status of children with diabetes [6].

The current study has several limitations that should be considered when interpreting the results. First, the sample size was relatively small, which may limit the generalizability of the findings to other populations. Secondly, the study was conducted at a single center, which may limit the external validity of the findings. Another limitation is the lack of multiple readings of HbA1c, which may have led to measurement errors and reduced the accuracy of the results.

## CONCLUSION

Patients with type 1 diabetes mellitus had significantly lower anthropometric measures than the general population. Furthermore, older children, female gender, large family size, and disease duration were independent predictors of undernutrition in T1DM. Moreover, there was a significant negative correlation between BMI and weight for age with metabolic control of diabetes as represented by HbA1c. Further multi-centric studies with a larger sample size should be carried out to strengthen the study results.
